# Neutrophil extracellular traps facilitate cancer metastasis: cellular mechanisms and therapeutic strategies

**DOI:** 10.1007/s00432-022-04310-9

**Published:** 2022-09-01

**Authors:** Wenxing Hu, Serene M. L. Lee, Alexandr V. Bazhin, Markus Guba, Jens Werner, Hanno Nieß

**Affiliations:** 1 Department of General, Visceral, and Transplant Surgery, University Hospital, Ludwig-Maximilians-University, Marchioninistr. 15, 81377 Munich, Germany; 2 German Cancer Consortium (DKTK), Partner Site Munich, Munich, Germany; 3 Bavarian Cancer Research Center (BZKF), Erlangen, Germany

**Keywords:** Neutrophil extracellular traps, NETs, Tumor growth, Cancer progression, Metastasis, Therapy

## Abstract

**Background:**

The formation of neutrophil extracellular traps (NETs) was initially discovered as a novel immune response against pathogens. Recent studies have also suggested that NETs play an important role in tumor progression. This review summarizes the cellular mechanisms by which NETs promote distant metastasis and discusses the possible clinical applications targeting NETs.

**Method:**

The relevant literature from PubMed and Google Scholar (2001–2021) have been reviewed for this article.

**Results:**

The presence of NETs has been detected in various primary tumors and metastatic sites. NET-associated interactions have been observed throughout the different stages of metastasis, including initial tumor cell detachment, intravasation and extravasation, the survival of circulating tumor cells, the settlement and the growth of metastatic tumor cells. Several in vitro and in vivo studies proved that inhibiting NET formation resulted in anti-cancer effects. The biosafety and efficacy of some NET inhibitors have also been demonstrated in early phase clinical trials.

**Conclusions:**

Considering the role of NETs in tumor progression, NETs could be a promising diagnostic and therapeutic target for cancer management. However, current evidence is mostly derived from experimental models and as such more clinical studies are still needed to verify the clinical significance of NETs in oncological settings.

## Introduction

Neutrophil extracellular traps (NETs) are web-like chromatin structures that are made up of a DNA backbone decorated with histones and granule proteins. They are released from neutrophils and were initially described by Brinkmann et al. in 2004 (Brinkmann et al. 2004). They reported NET formation as an innate immune response, which can trap and kill bacteria (Brinkmann et al. 2004). Since the discovery of NETs, more details of NETs have been uncovered. A growing body of evidence shows that NET formation occurs not only in infectious disease, but can also be triggered by other various stimuli (Table 1) and NETs play instrumental roles in various non-infectious conditions, including malignancy (Albrengues et al. 2018; Boone et al. 2015; Cools-Lartigue et al. 2013; Demers et al. 2016; Martins-Cardoso et al. 2020; Miller-Ocuin et al. 2019; Monti et al. 2018; Najmeh et al. 2017; Nie et al. 2019; Park et al. 2016; Rayes et al. 2019; Ren et al. 2021; Teijeira et al. 2020; Tohme et al. 2016; Xiao et al. 2021; Yang et al. 2020a, b; Yazdani et al. 2019; Zha et al. 2020), autoimmune disease (Apel et al. 2021; Carmona-Rivera et al. 2015; Kahlenberg et al. 2013; Parackova et al. 2020; Schauer et al. 2014), vascular disease (Binet et al. 2020; Grässle et al. 2014; Kang et al. 2020; Quillard et al. 2015; Warnatsch et al. 2015), surgical stress and traumatic injury (Cools-Lartigue et al. 2013; Liu et al. 2019; Ren et al. 2021; Tohme et al. 2016; von Meijenfeldt et al. 2018). Lytic NETosis and non-lytic NETosis are terms used to describe two distinct processes of NET formation (Pieterse et al. 2016). During lytic NETosis, the plasma membranes of neutrophils break down resulting in the release of NETs and subsequent cell death (Fuchs et al. 2007). In contrast, plasma membranes remain intact during non-lytic NETosis, in which neutrophils export NETs by releasing vesicles that contain DNA (Thiam et al. 2020). In this review, we will discuss the cellular mechanisms of NETosis and highlight NET-mediated actions during metastasis.

**Table 1:** Inducers of NET formation

NET-stimulating substances	Source of stimuli	Types of NETosis	Reference
PMA	–	Lytic	Brinkmann et al. (2004)
HMGB1 and other DAMPs	Tumor, radiotherapy, I/R injury, surgical stress	Lytic and non-lytic	Dyer et al. (2018); Kim et al. (2019); Ren et al. (2021); Shinde-Jadhav et al. (2021); Tadie et al. (2013); Zha et al. (2020)
IL-8 and CXCR1/2 agonists	Tumor cells	Lytic	Nie et al. (2019); Zha et al. (2020)
Cathepsin C	Tumor cells	Lytic (ROS-dependent)	Xiao et al. (2021)
G-CSF	Tumor cells	Unspecified	Demers et al. (2016)
Amyloid β	Cancer-associated fibroblasts	Lytic	Munir et al. (2021)
IL-1β, CXCL1	Senescent endothelial cells	Unspecified	Binet et al. (2020)
IL-1β and IL-18	Macrophage	Unspecified	Kahlenberg et al. (2013); Warnatsch et al. (2015)
LPS and other PAMPs	Pathogens (e.g. *S. aureus*, *E. coli*, *P. aeruginosa*)	Non-lytic (platelet-dependent); Lytic (ROS-dependent)	Brinkmann et al. (2004); Clark et al. (2007); Pieterse et al. (2016); Pilsczek et al. (2010); Yipp et al. (2012)
Chemicals in cigarette	Smoking	Unspecified	Albrengues et al. (2018); Qiu et al. (2017)
Bleomycin	Chemotherapy	Unspecified	Liu et al. (2019)
Monosodium urate	Gout	Non-lytic	Schauer et al. (2014)

*NET *neutrophil extracellular trap, *PMA* phorbol 12-myristate 13-acetate, *HMGB1* high mobility group box 1, *DAMPs* damage-associated molecular patterns, *I/R* ischemia/reperfusion, *IL* interleukin, *CXCR* CXC chemokine receptor, *CXCL1/2* chemokine ligand 1/2, *G-CSF* granulocyte-colony stimulating factor, *ROS* reactive oxygen species, *LPS* lipopolysaccharide, *PAMPs* pathogen-associated molecular patterns

## The mechanisms of NET formation

**Fig. 1:**
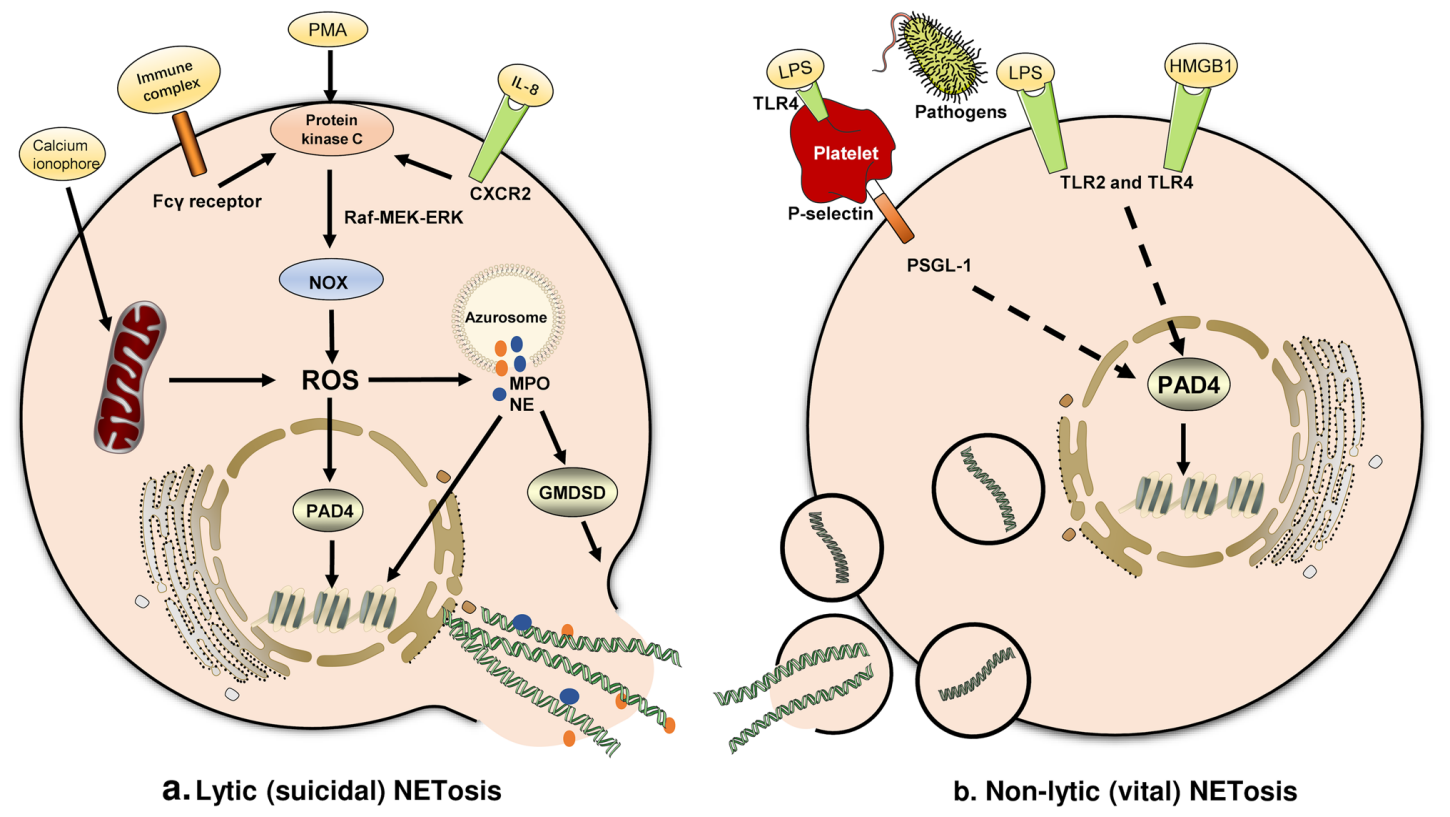
Mechanisms of lytic and non-lytic NETosis. **a** Mechanism of lytic NETosis. External stimuli, such as Phorbol 12-myristate 13-acetate (PMA), IL-8 and immune complex, could activate membrane-bound NADPH oxidase (NOX) to produce reactive oxygen species (ROS). Accumulation of ROS leads to the release of neutrophil elastase (NE) and myeloperoxidase (MPO) from azurosomes. NE and peptidylarginine deiminase 4 (PAD4) both contribute to histone modification and chromatin decompaction, which are followed by nuclear membrane breakage. Chromatin and nuclear proteins are then released into cytoplasmic space, where they interact with cytoplasmic proteolytic enzymes and antibacterial peptides. Gasdermin D (GSDMD) induces plasma membrane rupture and mediates the expulsion of NETs into the extracellular space. **b** Mechanism of non-lytic NETosis. Lipopolysaccharide (LPS) or HMGB1 could induce neutrophils to release NETs in a non-lytic manner through TLR2/4-mediated signaling pathways. Alternatively, TLR4-activated platelets could also predispose non-lytic NETosis through the interaction between P-selectin and its ligand, PSGL-1. Unlike lytic NETosis, non-lytic NETosis is ROS-independent and DNA-NETs are released via vesicular export without the rupture of plasma membrane

Lytic NETosis (Fig. 1a), also known as suicidal NET formation, is a special programmed cell death driven by reactive oxygen species (ROS). Various external stimuli, including crossing-linking of immune complex and Fcγ receptor (Alemán et al. 2016a, 2016b), interaction between interleukin-8 (IL-8) and C–X–C motif chemokine receptor 2 (CXCR2) (An et al. 2019; Nie et al. 2019; Zha et al. 2020), or direct stimulation by phorbol 12-myristate 13-acetate (PMA), are able to activate protein kinase C and lead to NADPH oxidase-dependent ROS production via the Raf-MEK-ERK signaling pathway (Gray et al. 2013; Hakkim et al. 2011). In some cases, ROS production may be NADPH oxidase-independent, for example, calcium ionophore-induced NETosis is mediated by calcium-dependent SK3 channel and relies on mitochondrial ROS production (Douda et al. 2015). The accumulation of intracellular ROS eventually leads to the rupture of azurophilic granules and the release of myeloperoxidase (MPO) and neutrophil elastase (NE). Myeloperoxidase and NE act synergistically to enhance their proteolytic activities and translocation into the nucleus (Branzk et al. 2014; Metzler et al. 2014; Papayannopoulos et al. 2010). Before reaching the nucleus, NE may cause degradation of the actin cytoskeleton, which leads to neutrophil immobilization and settlement of NETs within the foci of disease (Metzler et al. 2014). Once within the nucleus, NE could cleave histone H4 and unwind condensed chromatin. Besides NE-mediated histone modification, the citrullination of histones by peptidylarginine deiminase 4 (PAD4) is another mechanism that induces chromatin decompaction. Under the influence of Ca^2+^ and ROS (Rohrbach et al. 2012), PAD4 is activated and drives the conversion of arginine residues to citrullines in histone H3 and H4, which reduces the histones’ electrostatic attraction to DNA strands and promotes chromatin decondensation (Kaplan and Radic 2012). Chromatin swelling and nuclear lamin disassembly promotes the release of DNA into the cytoplasm (Li et al. 2020; Neubert et al. 2018), where cytoplasmic proteins could bind to DNA strands before the extrusion of NETs (Papayannopoulos et al. 2010). Gasdermin D (GSDMD), a pore-forming protein, which is activated by NE and caspase-4, mediates the final step of NETosis by inducing the expulsion of DNA into the extracellular space. Activated GSDMD not only forms pores in the plasma membrane, but also forms pores in the granule membrane and results in the release of more NE, which significantly enhances NET formation by establishing a positive-feedback loop (Sollberger et al. 2018).

In 2007, Clark et al. reported that interactions between platelets and neutrophils could induce NETosis without resulting in neutrophil death (Clark et al. 2007). ﻿When neutrophils release NETs under the stimulation of platelets, their plasma membrane integrity was maintained (shown by lack of staining by cell-impermeant nuclear dye Sytox Green). Later this process was named as non-lytic NETosis or vital NETosis (Fig. 1b). Most host-defense functions, such as phagocytosis, remain intact in neutrophils undergoing non-lytic NETosis (Yipp et al. 2012). Unlike lytic NETosis, which usually takes several hours to occur, non-lytic NETosis happens much faster. It has been documented that several pathogens, including *S. aureus* and *C. albicans*, could promote vital NETosis by activating TLR2 or C3a receptors (Yipp et al. 2012). Additionally, the crosstalk between neutrophils and platelets, which are activated by the LPS-TLR4 axis, also contributes to non-lytic NETosis (Clark et al. 2007; Pieterse et al. 2016). Although the molecular mechanism of non-lytic NETosis remains unclear, Pilsczek et al. have pointed out that DPI, a NADPH oxidase blocker, failed to inhibit *S. aureus*-induced NETosis over the first hour, which suggests that non-lytic NETosis may be NOX-independent (Pilsczek et al. 2010).

﻿﻿Due to the diversity of upstream signaling pathways, the same stimulant may result in different types of NETosis. For example, LPS derived from *P. aeruginosa* and *E. coli O128:B12* often induces ROS-dependent lytic NETosis (Pieterse et al. 2016). However, when neutrophils are co-cultured with platelets, LPS released by *P. aeruginosa and E. coli O111:B4* is more likely to induce vital NETosis (Pieterse et al. 2016). The type of NETosis may also change over time. When neutrophils are exposed to *S. aureus*, non-lytic NETosis predominates in the early phase, but is replaced by lytic NETosis in the late stage. The content of non-lytic and lytic NETs may differ depending on the time of release and cell membrane integrity. Despite both mechanisms having bactericidal effects, non-lytic NETs have significantly lower protease content than lytic NETs (Pieterse et al. 2016; Pilsczek et al. 2010). NET-bound proteases, such as NE and MPO, are potent inducers of several NET-mediated responses (Albrengues et al. 2018; Chen et al. 2021; Yazdani et al. 2019). These two difference types of NETosis may serve different functions, the rapid release of NETs without neutrophil destruction could allow timely neutralization of pathogens. Whereas the slow release of NETs in a lytic manner, containing more bioactive compounds, may lead to or exacerbate inflammation and influence the progression of chronic disease.

## The functional components of NETs and their mechanisms of action

In addition to their DNA backbone, NETs could have more than one hundred possible proteins as their constituents (Lim et al. 2018; Urban et al. 2009). Based on their origin and function, the most commonly found NET proteins can be classified into the following categories: nuclear proteins (e.g. citrullinated histone, HMGB1), antimicrobial and pro-inflammatory peptides (e.g. cathelicidin, calprotectin, lactotransferrin, complement C3), cytoskeletal elements (e.g. cytokeratin, actin), proteolytic enzymes (e.g. NE, MMP, cathepsin G, lipocalin-2), and other metabolic enzymes (e.g. transketolase, enolase). The interactions between DNA and bound proteins largely amplify the bioactivities of NETs. Extracellular DNA of NETs could protect bound proteins from denaturation, preserve the biological functions of proteins (Hahn et al. 2019; Papayannopoulos et al. 2010; Saffarzadeh et al. 2012) and serve as a proteolysis scaffold (Albrengues et al. 2018). The NET-bound proteins in turn protect DNA from nuclease degradation (Neumann et al. 2014) and enhance DNA recognition (Apel et al. 2021; Garcia-Romo et al. 2011). Because of the diversity of components, NETs can be recognized by a variety of cellular receptors which lead to the activation of different signaling pathways. The receptors for NET components and their biological actions are summarized in Table 2.

**Table 2:** Actions mediated by NETs

Actions/effects	Functional components of NETs	Target cells	Target molecules/receptors	Mechanism	Reference
Promote tumor growth in established liver micrometastases	HGMB1	Metastatic CRC cells	TLR9	TLR9-dependent pathways	Tohme et al. (2016)
Promote proliferation and invasion of glioma cells	HMGB1	Glioma cells	RAGE	HMGB1/RAGE axis	Zha et al. (2020)
Promote distant metastasis by inducing migration, adhesion and proliferation of tumor cells	DNA	Metastatic breast and colorectal cancer cells	CCDC25	CCDC25-ILK-β parvin-RAC1 signaling	Yang et al. (2020a)
Promote tumor growth by enhancing their mitochondrial biogenesis and energy production	Neutrophil elastase	Metastatic CRC cells	TLR4	TLR4-p38-PGC1α signaling (PGC1α is a transcriptional coactivator that regulates genes involved in energy metabolism)	Yazdani et al. (2019)
Awaken dormant cancer cells in the lung and promote tumor growth	Neutrophil elastase, MMP9	Dormant breast cancer cell	Laminin	DNA backbone of NETs serves as a proteolysis scaffold, which facilitates NET-associated proteases to process laminin-111. NET-remodeled laminins awaken dormant cancer cells through integrin α3β1 signaling	Albrengues et al. (2018)
Promote proliferation and migration of diffuse large B-cell lymphoma	IL-8 induced NETs	DLBCL Lymphoma cells	TLR9	TLR9-dependent signaling	Nie et al. (2019)
Increase the invasion and migration of tumor cells	CXCL1/2-induced NETs	Breast cancer cells	Unspecified	Unspecified	Park et al. (2016)
Promote EMT in breast tumor cells	NETs (unspecified)	Breast cancer cells	Unspecified	Upregulation of pro-inflammatory genes and EMT-associated genes in tumor cells	Martins-Cardoso et al. (2020)
Enhance the cytotoxicity resistance and invasion of HCC cells	NETs (unspecified)	HCC tumor cancer cells	TLR4 TLR9	Upregulation of inflammatory mediators in tumor cells through TLR4/9-COX2 signaling	Yang et al. (2020b)
Promote adhesion of circulating tumor cells	β1-integrin on NETs	Metastatic lung cancer cells	β1-integrin on tumor cells	β1 integrin on both sides may be connected through a bridging molecule (e.g. ECM proteins)	Najmeh et al. (2017)
Promote tumor adhesion by trapping circulating tumor cells within liver sinusoid	Inflammation-induced NETs	Metastatic lung cancer cells	Unspecified	Unspecified (possibly mediated by interaction between β1-integrin on NETs and ICAM-1 on tumor cells)	Cools-Lartigue et al. (2013)
Promote lung metastasis of breast cancer cells	NET-associated proteases	Metastatic breast cancer cells	TSP-1	NET-associated proteases degrade TSP-1, which is a key metastasis-suppressive ECM protein	Xiao et al. (2021)
Limit immune-mediated cytotoxicity to tumor cells	Tumor-induced NETs	Lymphocyte Tumor cells	N/A	NETs provide a physical shield to prevent lymphocytes/NK cells from reaching tumor cells	Teijeira et al. (2020)
Promote the proliferation of lung fibroblasts and their differentiation into myofibroblasts	CpG motifs of DNA, MPO, histone	Lung fibroblast (in interstitial lung diseases)	TLR9	TLR9‐miR‐7‐Smad2 pathway	Chrysanthopoulou et al. (2014); Zhang et al. (2020b)
Activate pancreatic stellate cells and enhance pancreatic tumor growth	DNA	Pancreatic stellate cells	RAGE	RAGE-dependent signaling	Miller-Ocuin et al. (2019)
Contribute to pathogenesis of SLE through inducing type I interferon production by macrophages	DNA	Macrophages	Intracellular sensor cyclic GMP-AMP synthase (cGAS)	DNA-cGAS-STING signaling	Apel et al. (2021)
Impair phagocytic clearance of apoptotic cells by the macrophages (efferocytosis) in sepsis	Neutrophil elastase	Macrophage	Integrin α_v_β_3_ and α_v_β_5_	Efferocytosis is mediated by MFG-E8 on apoptotic cells and its receptor integrin α_v_β_3_/α_v_β5 on macrophages. NET-associated NE impedes efferocytosis through disruption of α_v_β_3_/α_v_β_5_ integrins	Chen et al. (2021)
Induce hepatocyte death and augment cytokine production by Kupffer cells	Liver I/R-induced NETs	Hepatocyte Kupffer cell	Unspecified	Unspecified	Huang et al. (2015)
Induce epithelial and endothelial cell death	Histone, MPO	Alveolar epithelial cell and endothelial cell	Unspecified	Unspecified	Saffarzadeh et al. (2012))
Disrupt the integrity of the intestinal barrier, thus exacerbate sepsis	Sepsis-induced NETs	Intestinal epithelial cell	TLR9	TLR9-mediated endoplasmic reticulum stress pathway	Sun et al. 2021)
Induce EMT of alveolar cells	COVID-induced NETs	Alveolar epithelial cells	Unspecified	Unspecified (possibly facilitated by alveolar macrophage)	Pandolfi et al. (2021)
Lower the activation threshold of T lymphocytes	NETs (unspecified)	T lymphocyte	TCR-mediated	NETs do not have a direct effect on T cell activation. However, NETs may lower the activation threshold of T cells, making them more easily activated by other cells, e.g. monocyte-derived dendritic cells	Tillack et al. (2012)
Induce Th-cell-mediated immune response and promote chronic inflammation in smoking and type-1 diabetes	NETs (unspecified)	Plasmacytoid dendritic cells, Naïve T cells	Unspecified	NETs-activated pDCs induce naïve T cell differentiation into Th1 and Th17 cell	Parackova et al. (2020); Qiu et al. (2017)

*NET *neutrophil extracellular trap, *TLR* toll-like receptor, *HMGB1* high mobility group box 1, *RAGE* receptor for advanced glycation endproducts, *CRC* colorectal cancer, *DLBCL *diffuse large B-cell lymphoma, *CXCL* CXC chemokine ligand,  *EMT* epithelial–mesenchymal transition, *HCC *hepatocellular carcinoma, *TSP-1* thrombospondin-1, *ECM* extracellular matrix, *MMP* matrix metalloproteinases, *MPO*, myeloperoxidase, *SLE* systemic lupus erythematosus, *I/R* ischemia/reperfusion, *STING* stimulator of interferon genes, *pDCs* plasmacytoid dendritic cells

Pattern recognition receptors (PRRs) are a class of host sensors that recognize both exogenous pathogen-associated molecular patterns (PAMPs) and endogenous damage-associated patterns (DAMPs). NETs are deemed as DAMPs and are recognized by the vast majority of pattern recognition receptors (PRRs). Toll-like receptors (TLRs), a family of PRRs widely expressed on innate immune cells and non-immune cells, are extensively studied in NET-mediated response. Several proteins on NETs, such as NE and histones, can activate plasma membrane-located TLR2 and TLR4. Once NETs are taken up by target cells, the cytoplasmic receptor TLR9 can recognize CpG motifs of DNA-NETs. Activation of TLRs and their downstream MyD88-dependent signaling pathways then induce pro-inflammatory changes in target cells, which exacerbates disease progression. Exposure to the DNA-peptide complex can provoke intracellular signaling more easily than exposure to DNA alone with the best example being cathelicidin on NETs. In the context of autoimmune diseases, such as psoriasis and SLE, the combination of cathelicidin and DNA enables inert DNA to become a potent TLR9 activator that induces cytokine production in plasmacytoid dendritic cells (Garcia-Romo et al. 2011; Lande et al. 2011, 2007). Similar NET-induced changes were noted in macrophages, in which cathelicidin-NET complexes activate NLPR3 inflammasomes and lead to the release of IL-1β and IL-18 (Kahlenberg et al. 2013; Warnatsch et al. 2015). Secreted IL-18 could further promote NETosis and amplify the inflammation response.

The receptor for advanced glycation end products (RAGE) is another transmembrane receptor which serves as PRRs (Sparvero et al. 2009). Several constituents of NETs, such as HMGB1 and S100 proteins, are considered RAGE ligands (Sparvero et al. 2009). RAGE plays an important role in both NET formation and NET-mediated response (Boone et al. 2015; Miller-Ocuin et al. 2019). HMGB1 on NETs was reported to activate RAGE and trigger downstream NF-κB signaling in glioblastoma (Zha et al. 2020), while DNA-NETs were found to be responsible for RAGE-dependent activation of pancreatic stellate cells (Miller-Ocuin et al. 2019).

Similar to TLR9, cyclic GMP-AMP synthase (cGAS) is intracellular DNA sensor which can also recognize phagocytosed NETs. The activation of NETs-mediated cGAS-STING pathways prime macrophages to type I interferon production and contribute to the pathogenesis of several diseases (Apel et al. 2021; Kang et al. 2020). Besides PRRs and cGAS, NETs can also interact with CDCC25, a transmembrane protein, which was poorly understood in the past. It was recently discovered that through stimulation with DNA-NETs, CCDC25 on tumor cells could initiate the ILK-β parvin signaling pathway to remodel the intracellular cytoskeleton, which facilitates migration and adhesion of tumor cells (Yang et al. 2020a).

Aside from binding to cellular receptors, NETs also take advantage of attached proteases to elicit cellular response. As examples, NET-bound elastase can prevent macrophages from efferocytosis of apoptotic cells by disruption of αvβ3/αvβ5 integrins (Chen et al. 2021). NET-bound cathepsin G could process pro-IL-1α to IL-1α, a powerful inducer of adhesion molecules and tissue factors on endothelial cells (Folco et al. 2018).

## NETs and cancer progression

**Fig. 2:**
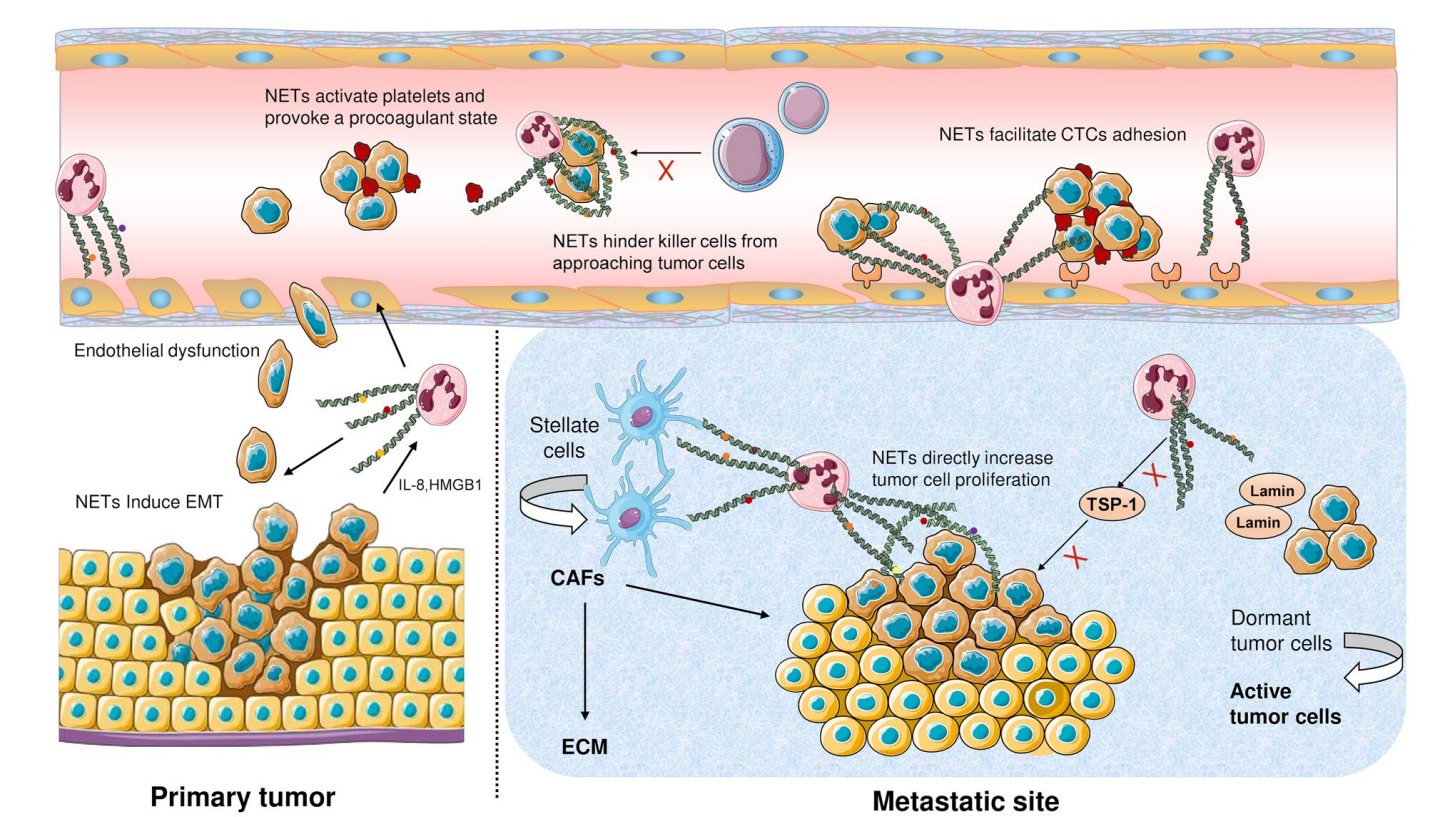
NETs promote tumor growth and distant metastasis. In primary tumor, the release of IL-8 and HMGB1 predisposes NET formation, which induces epithelial–mesenchymal transition (EMT) and promote tumor cell proliferation, migration, and invasion of tumor cells. NETs also contribute to local endothelial–mesenchymal transition and endothelial dysfunction, eventually facilitating the intravasation of tumor cells. During dissemination, NETs may protect circulating tumor cells (CTCs) from cytotoxic attacks of immune cells. NETs may also activate platelets and provoke a procoagulant state. Infection and inflammation could induce the release of NETs in the distant organs, leading to potential upregulation in the expression of endothelial adhesion molecules. Once CTCs arrive the host organ, the pre-existing NET formation could mediate the adhesion and settlement of CTCs. Apart from directly promoting tumor cell proliferation, NETs could induce metastatic tumor growth by recruiting tumor stromal cells such as cancer-associated fibroblasts (CAFs). Furthermore, NETs could modify the extracellular matrix (ECM) to enhance distant metastasis. For instance, NET-remodeled laminin awakens dormant tumor cells, while degradation of thrombospondin-1 (TSP-1) minimizes its inhibitory effects on tumorigenesis

There is increasing evidence that tumor-derived substances, including IL-8 and HMGB1, could induce NET formation (Nie et al. 2019; Ren et al. 2021; Zha et al. 2020). NETs eventually contribute to the tumor growth and distant metastasis (Fig. 2). The presence of intra-tumoral NETs were confirmed by immunofluorescence staining in multiple cancer tissues from mice and humans (Guan et al. 2021; Oklu et al. 2017; Park et al. 2016; Yang et al. 2020a, b). Furthermore, markers of circulating NETs, such as H3Cit and MPO-dsDNA, are found to be elevated in patients with lung metastases (Yang et al. 2020a) and colorectal liver metastases (Tohme et al. 2016). To some extent, the circulating NETs may reflect the severity of the malignant disease. In HCC patients, H3Cit-DNA level was significantly higher in HCC patients with tumors ≥ 8 cm (Zenlander et al. 2021). A similar study showed that the level of MPO-dsDNA was much higher in patients with advanced stage esophagogastric or lung adenocarcinoma than those with low stage carcinoma (Rayes et al. 2019).

The occurrence of NETosis may precede the arrival of circulating tumor cells. NETs are detected in various conditions other than malignancies, such as local infection, chronic obstructive pulmonary disease (Dicker et al. 2018), steatohepatitis (van der Windt et al. 2018), liver cirrhosis (Zenlander et al. 2021) and surgical stress (Cools-Lartigue et al. 2013; Ren et al. 2021; von Meijenfeldt et al. 2018). The pre-existing NETs may shape a pre-metastatic niche to host the arriving tumor cells, for example, liver ischemia reperfusion (I/R) injury models demonstrated that surgical stress could result in widespread NETosis (Zhang et al. 2020a), NETs deposited in the lung microvasculature could capture circulating tumor cells and promote distant metastasis (Ren et al. 2021).

### NETs promote the epithelial–mesenchymal transition of cancer cells

Epithelial–mesenchymal transition (EMT) is a cellular programme involved in embryogenesis, tissue regeneration, wound healing, and metastasis. Key features of EMT include the disruption of cell–cell and cell–matrix adhesion, the loss of cellular polarity, and cytoskeletal remodeling (Dongre and Weinberg 2019). This transition is adopted by tumor cells to enable migration and invasion. Recent studies demonstrated that NETs and their components are able to induce EMT in various types of tumor cells, including breast cancer cells (Martins-Cardoso et al. 2020), gastric cancer cells (Zhu et al. 2021) and pancreatic cancer cells (Jin et al. 2021; Kajioka et al. 2021). After exposure to NETs in vitro, tumor cells developed morphology alterations, which are characterized by elongated fibroblast-like shape and the loss of adhesion to the flask wall. Immunoblotting showed reduced expression of epithelial markers, such as E-cadherin, and enhanced expression of mesenchymal markers, such as N‐cadherin, vimentin and α-SMA (Kajioka et al. 2021; Zhu et al. 2021). The mRNA levels of EMT regulators, including ZEB, SLUG and SNAIL, are also upregulated in NET-treated tumor cells. These transcriptional and translational alterations observed in NET-induced EMT are similar to the changes in TGF-β-induced EMT (Katsuno et al. 2013). NET-induced EMT and related tumor progression could be inhibited in vivo by administration of NET inhibitors, DNase and PAD4 blocker (Zhu et al. 2021). Nevertheless, the exact mechanism behind NET-induced EMT are still unclear. Kajioka et al. believed that NET-bound HMGB1 is the main activator of NET-induced EMT. Similar findings were observed when pancreatic tumor cells were treated with free HMGB1 and NET-induced EMT was blocked when anti-HMGB1 thrombomodulin was applied (Kajioka et al. 2021). However, Jin et al. and Martins-Cardoso et al. suggested that NET-mediated pro-inflammatory response and upregulation of inflammatory cytokines are responsible for EMT changes (Jin et al. 2021; Martins-Cardoso et al. 2020). It is worth noting that NET-induced EMT is not only found in cancerous conditions*. *In vitro airway models and lung biopsies of deceased patients suggest that NETs may also contribute to the EMT of alveolar epithelial cells and lung fibrosis in COVID-19 (Pandolfi et al. 2021).

### NETs may facilitate the intravasation and extravasation of cancer cells

Metastasis requires the entry of tumor cells into the circulation (intravasation) and their exit from the circulation (extravasation) into host tissue. Multiple strategies are adopted by tumor cells to cross the endothelial barrier during these processes. The EMT described above allows tumor cells to abrogate cell–cell adhesion and pass through the endothelial junctions in an advantageous shape. In addition to promoting epithelial–mesenchymal transition in tumor cells, NETs also contribute to endothelial-to-mesenchymal transition. VE-cadherin/β-catenin is an adherens junction complex which maintains the integrity of the endothelial barrier. Degradation of cadherin by NET-bound elastase breaks down the adhesion between endothelial cells. Undocked intracellular β-catenin then translocates into the nucleus and subsequently activates the EMT-associated signaling pathway. Treatment with NETs significantly increases vascular leakage and induce proteinuria in vivo (Pieterse et al. 2017). Similar studies have shown that NETs disrupt the integrity of the blood–brain barrier during stroke (Kang et al. 2020), resulting in increased permeability and delayed vascular damage. The endothelial barrier is sustained by endothelial cells and adhesion molecules; injury of endothelial cells leads to barrier dysfunction and facilitates trans-endothelial migration of tumor cells. One previous study reported that tumor cells can promote trans-endothelial migration by inducing endothelial necroptosis (Strilic et al. 2016). In fact, several studies have confirmed that NETs directly contribute to endothelial cell apoptosis (Binet et al. 2020; Gupta et al. 2010; Saffarzadeh et al. 2012), which can cause a breach that may provide a gateway for tumor cell passage. NETs contain several collagenases, such as MMP2 and MMP9, which might further decrease cell adherence and increase damage to the endothelial barrier by degrading basement membrane proteins (Quillard et al. 2015). In addition, NETs may enhance the motility of tumor cells by inducing cytoskeleton rearrangement via the CCDC25-ILK signaling pathway (Yang et al. 2020a), which may further facilitate tumor cell transmigration across the endothelium.

### A potential role of NETs in inducing a procoagulant state and escorting circulating tumor cells to the metastatic niche

When tumor cells leave the primary site of malignancy to circulate in blood and lymph, they are at high risk of being killed by cytotoxic immune cells and the shear stress generated by blood flow (Regmi et al. 2017). To increase the chance of survival, circulating tumor cells (CTCs) either travel as cell clusters (Aceto et al. 2014) or recruit platelets and fibrins to form tumor cell-platelet aggregates (Anvari et al. 2021; Egan et al. 2014; Heeke et al. 2019). The attached platelets and fibrins not only provide an immune cell-resistant physical shield (Palumbo et al. 2005), but also promote adhesion of the CTCs to endothelium when they arrive the metastatic foci (Anvari et al. 2021).

Although no specific studies have confirmed the relationship between NETs and tumor cell-platelet aggregates, there is evidence that NETs are able to induce a procoagulant state, which may favor the formation of tumor cell-platelet aggregates. NETs (Elaskalani et al. 2018) and NET-associated molecules, such as cell-free DNA (Jansen et al. 2017), extracellular histones (Semeraro et al. 2011) and granular enzymes (Kolarova et al. 2013), were reported to trigger platelet activation and aggregation. Furthermore, NETs could provide a scaffold for the aggregation of platelets and other coagulation proteins (Fuchs et al. 2010), induce overexpression of tissue factor on endothelial cells (Folco et al. 2018; Haubitz et al. 2001; Yang et al. 2016) and inhibit fibrinolysis by degradation of plasminogen (Cruz et al. 2019). In fact, several studies have pointed out that tumor-induced NET formation plays an important role in cancer-associated thrombosis (Abdol Razak et al. 2017; Demers et al. 2012; Hisada et al. 2020; Mauracher et al. 2018).

Platelets and fibrins encoat and wrap around the CTCs, hence support their survival by impeding immune cell-mediated cytotoxicity (Palumbo et al. 2005). Likewise, CTCs have been noted to benefit from a similar protection bestowed by NETs. Teijeira et al. observed that when tumor spheroids were co-cultured with NK cells or CD8^+^ T cells (Teijeira et al. 2020), NETs-coated tumor spheroids grew much larger than those without NETs coating. Subsequent in vivo experiments further confirmed that NETs could impair the contact between NK cells and circulating tumor cells in liver sinusoids, and reduce CD8^+^ T cell infiltration into subcutaneous tumor. The inhibition of NET formation could restore the cytotoxic abilities of NK cells and reduce metastasis. Taking into account all the above findings, the coverage of NETs might protect circulating tumor cells from cytotoxic attacks during dissemination and settlement.

### NETs enhance the adhesion of circulating tumor cells

If CTCs survive the perils of dissemination, they eventually attach to the walls of the microvasculature and start extravasation. The adhesion of CTCs to endothelium is not an easy process due to a rapid and turbulent blood flow. The pre-existing NETs within metastatic target organs might facilitate adhesion by capturing CTCs (Najmeh et al. 2017; Ren et al. 2021).

As mentioned earlier, inflammation could promote the formation of NETs (Albrengues et al. 2018; Cools-Lartigue et al. 2013). Mouse models subjected to cecal ligation puncture will develop extensive abdominal septic response and deposit a massive amount of NETs within hepatic sinusoids. These intravascular NETs will then bind circulating tumor cells in an integrin-dependent manner (Najmeh et al. 2017). Najmeh et al. found that β1-integrin was present both on tumor cells and NETs and they hypothesized that these common subunits might be connected through a bridging molecule, such as an ECM protein (Najmeh et al. 2017). As the expression of integrins may vary depending on the type of tumor cells, the abilities of different tumor cells to adhere to NETs were tested in vitro in another study. This study showed that the expression level of integrins α5β1, αvβ3, and αvβ5 is positively correlated with the adhesion of tumor cells to NETs (Monti et al. 2018). Moreover, NETs have been shown to be able to capture tumor-platelet aggregates in a hepatic I/R injury model (Ren et al. 2021), possibly through interactions between NETs and TLR4-activated platelets (Elaskalani et al. 2018; Semeraro et al. 2011).

A further study indicated that NETs could induce upregulation of adhesion molecules on endothelium. Cathepsins bound to NETs are able to process pro-IL-1α into active form IL-1α, which then increases the expression of VCAM-1 and ICAM-1 in human endothelial cells (Folco et al. 2018). Overexpression of ICAM-1 in liver sinusoidal endothelial cells not only facilitates the recruitment of leukocytes and monocytes, but also enhances tumor cell adhesion and angiogenesis (Benedicto et al. 2019).

### NETs awaken dormant cancer cells and promote tumor growth

It is hypothesized that tumor dormancy is a strategy employed by metastatic tumor cells when they encounter a metastasis-resistant microenvironment. These quiescent cancer cells can be reactivated in response to stimuli and lead to the formation of macrometastasis. Recently, a study suggested that inflammation-induced NETs could awaken dormant breast cancer cells (Albrengues et al. 2018). In this study, NETosis was triggered by the exposure to nasal LPS instillation and tobacco smoke in a murine model. NET-bound proteases, NE and MMP9, were found to process the ECM protein laminin using NETs as a proteolysis scaffold. NET-remodeled laminins became more bioactive and bound integrin α3β1 expressed on dormant cancer cells, which drove awakening of cancer cells via FAK/ERK/MLCK/YAP signaling cascades. Moreover, the same study also showed that NETs facilitated the proliferation of metastatic breast and prostate cancer cells. In fact, there is a considerable amount of literature suggesting that NETs could directly promote the growth of tumor cells (Demers et al. 2016; Park et al. 2016).

Activation of PRR-mediated signaling is the most common strategy adopted by NETs to promote tumor growth. In diffuse large B-cell lymphoma cells (Nie et al. 2019) and colon adenocarcinoma cells (Tohme et al. 2016), NETs could exert pro-tumorigenic effects by activating TLR9 and its downstream signaling through pathways such as NF-kB and MAPK. Similar findings were noted in glioblastoma cells (Zha et al. 2020), in which NET-derived HMGB1 bound to RAGE and upregulated the NF-κB signaling. Another example is in hepatocellular carcinoma, where the activation of TLR4 and TLR9 by NETs led to upregulation of COX2, a potent inflammatory mediator, which enhanced the invasiveness of HCC cells (Yang et al. 2020b). In addition to provoking pro-inflammatory responses in tumor cells, NETs also alter the metabolism of tumor cells. PGC1α is a transcriptional coactivator that regulates the genes involved in energy metabolism. NETs could activate TLR4-mediated p38-PGC1α signaling pathway, which causes increased mitochondrial biogenesis in metastatic CRC cells and eventually contributes to tumor growth (Yazdani et al. 2019).

### NETs facilitate the remodeling of cancer-associated stroma

The tumor stroma consists of non-malignant cells (e.g. fibroblasts, immune cells and endothelial cells) and non-cellular components (e.g. extracellular matrix and basement membrane). The shaping of pro-tumorigenic stroma is critical for tumor progression and metastasis (Valkenburg et al. 2018). The extracellular matrix (ECM) is secreted by stromal cells and surrounds resident cells in tissues. The major ECM components include collagen, glycoproteins and proteoglycans. The composition and organization of these ECM elements largely affect cell signaling, cell migration, tumor growth and progression (Winkler et al. 2020). Increased deposition of adhesive glycoproteins, such as tenascin and fibronectin, favor tumor cell migration and invasion. In contrast, thrombospondin-1 (TSP-1) is an ECM protein which inhibits tumor growth and angiogenesis. Xiao et al. found that NET-bound proteases could degrade TSP-1 and thus support the metastatic growth of tumor cells (Xiao et al. 2021). Aforementioned NET-mediated modification of laminin is another example suggesting that NETs could process ECM proteins to promote tumorigenesis.

Cancer-associated fibroblasts (CAFs) are one of the most abundant non-malignant cells found in the tumor stroma. CAFs not only produce ECM proteins and soluble factors, but also release enzymes (e.g. lysyl oxidase) to process ECM components (Bremnes et al. 2011). In liver and pancreas, residing stellate cells are the primary source of CAFs. Recent studies found that pancreatic ductal adenocarcinoma-induced NETs could promote the activation of pancreatic stellate cells (Miller-Ocuin et al. 2019) and hepatic stellate cells (Takesue et al. 2020). These NET-activated stellate cells are closely correlated to the growth of primary tumor and the occurrence of liver metastasis, while degradation of NETs by DNase I inhibits the recruitment of CAFs and diminishes liver metastases (Miller-Ocuin et al. 2019; Takesue et al. 2020). The effect of NETs on fibroblasts has also been verified in non-cancerous conditions. It was reported that NETs-activated lung fibroblasts and led to exacerbation of pulmonary fibrosis (Chrysanthopoulou et al. 2014; Zhang et al. 2020b) NETs also induced the differentiation of monocytes to activated fibroblasts and promoted fibrous vascular occlusions (Sharma et al. 2021). Furthermore, substances derived from fibroblasts may amplify NET formation. For example, amyloid β secreted by CAFs can drive NET formation within primary murine pancreatic, skin and lung tumors (Munir et al. 2021). Therefore, NETs and fibroblasts may form a positive-feedback loop to enhance tumor growth and disease progression.

Tumor angiogenesis can be divided into three major parts: the inflammatory phase, the proliferative phase and the remodeling phase (Whipple and Korc 2010). During the inflammatory phase, leukocytes and monocytes are recruited to the tumor, where they produce pro-angiogenic factors to guide the proliferation of endothelial cells and stromal cells. Tumor-associated vessels are formed either by sprouting from pre-existing vessels locally or by recruiting endothelial progenitor cells from bone marrow (Chouaib et al. 2010). The early stage of tumor angiogenesis is characterized by destabilization and hyperpermeability (Whipple and Korc 2010). As discussed above, NETs may intensify and accelerate this process by inducing endothelial cell dysfunction and local basement membrane degradation. Furthermore, NETs could directly exert a pro-angiogenic activity on endothelial cells (Aldabbous et al. 2016; Jung et al. 2019; Yuan et al. 2020). Aldabbous et al. found that the stimulation of NETs could significantly promote the proliferation of human pulmonary artery endothelial cells, as shown by enhanced in vitro tube formation and spheroid sprouting, as well as increased plug vascularization in vivo (Aldabbous et al. 2016). The pro-angiogenic effect of NETs is also reflected in tumor angiogenesis. Mice receiving co-implantation of HCC cells and NETs showed a stronger expression of CD31, a reliable and widely adopted angiogenic marker, compared with mice implanted with HCC cells alone (Yang et al. 2020b). In another similar study, liver ischemia–reperfusion injury was introduced to induce NET formation before the injection of MC38 tumor cells. The tumor growth and expression of CD31 were largely reduced when the mice were also treated with NETs inhibitor DNase, compared to non-treated group (Tohme et al. 2016).

### The interaction between NETs and immune cells

Elevated neutrophil-to-lymphocyte ratio and increasing neutrophil infiltration have been linked to poor prognosis in various malignancies, including colorectal cancer (Halazun et al. 2008), hepatocellular carcinoma (Margetts et al. 2018) and breast cancer (Corbeau et al. 2020). Apart from releasing NETs, infiltrating neutrophils are able to exert multiple pro-tumorigenic effects (Wang et al. 2018). Interestingly, recent study showed that NETs could interact with neutrophils to form a positive-feedback loop. NETs upregulate β2 integrin expression on neutrophils (which bind onto endothelial ICAM-1), and promote the translocation of P-selectins on endothelial cells (which interact with neutrophil PSGL-1) (Lavoie et al. 2018). These two concurrent changes significantly facilitate the rolling and adhesion of leukocytes. Furthermore, NETs may modulate the recruitment of immune cells by adjusting the concentration of local inflammatory mediators. Researchers have found that NET-associated proteases may selectively degrade cytokines and chemokines (Hahn et al. 2019; Schauer et al. 2014), including IL-1β, IL-6, monocyte chemoattractant protein-1, macrophage inflammatory proteins and TNF. In contrast, the IL-8, a potent neutrophil attractant and NETs stimulator, is degraded to a lesser extent (Schauer et al. 2014). In fact, several studies have pointed out that NETs could upregulate IL-8 expression and secretion (Dömer et al. 2021; Hudock et al. 2020; Zha et al. 2020), which might attract more neutrophils to deposit additional NETs in disease foci. In addition to playing a role in the recruitment of neutrophils, NETs may further boost the pro-inflammatory activities of neutrophils, such as promoting their ROS production and phagocytosis (Dömer et al. 2021).

CD8^+^ T cells are important effector lymphocytes responsible for the anti-cancer immune response. Checkpoint molecules, such as CTLA4 and PD-1, are immune inhibitory proteins which act as an "off switch" to prevent T cells from attacking other cells. As such, checkpoint blockade immunotherapy could enhance the cytotoxic effect of T cells on tumor cells. Recent studies found that NETs are associated with resistance to checkpoint inhibitor treatment in pancreatic cancer (Zhang et al. 2020c) and colorectal cancer (Zhang et al. 2021). Inhibition of NETs by systemic administration of DNase I or the use of a PAD4^−/−^ model, largely improves the efficacy of﻿ anti-PD-1 immunotherapy, which is manifested by reduced tumor growth, increased tumor-associated CD8^+^ T cell infiltration and cytotoxicity. A previous study has pointed out that NETs may act as a barrier to access and prevent cytotoxic immune cells from approaching tumor cells effectively (Teijeira et al. 2020), which may explain why NETs cause resistance to anti-PD-1 therapy.

Although little knowledge exists about the crosstalk between NETs and tumor-associated immune cells, the impact of NETs on immune cells has been well documented in the context of autoimmune disease. A series of studies have revealed that NETs contribute to the pathogenesis of autoimmune disease by interacting with local immune cells, including macrophages (Krishnamoorthy et al. 2018; Warnatsch et al. 2015), plasmacytoid dendritic cells (Garcia-Romo et al. 2011; Parackova et al. 2020; Qiu et al. 2017; Villanueva et al. 2011) and T lymphocytes (Lambert et al. 2019; Tillack et al. 2012). For example, NETs can accelerate the disease progression of SLE by through the stimulation of macrophages and the resultant upregulation of the STING pathway, which is also extensively involved in tumor growth and metastasis (Ahn et al. 2014; Lemos et al. 2016; Liang et al. 2015; Song et al. 2017). It is very likely that these findings, regarding NET-triggered signaling pathways discovered in inflammation, could also be applicable in the scenario of carcinogenesis.

## The potential therapeutic applications of NETs

Since NETs play an influential role in tumor growth and metastasis, targeting NETs could be a promising and novel approach against cancer. A number of pre-clinical models have demonstrated that inhibiting NETosis or inhibiting NET-mediated action could improve disease progression. In the following section, we will discuss how these NET inhibitors (Table 3) can be applied into clinical practice.

**Table 3:** NET inhibitors

Type	Compound	Effect	Type of study	Reference
CXCR1 and CXCR2 antagonist	Reparixin SCH527123	Reduce lytic NETosis	In vitro	Teijeira et al. (2020); Ashar et al. (2021)
CXCR2 antagonist	SB225002,AZD5069	Reduce lytic NETosis	In vitro	Pedersen et al. (2018); Zha et al. (2020)
LPS neutralizing agent	Taurolidine	Decrease smoke-induced NETosis and attenuate NET-related activation of quiescent cancer cells	In vivo	Albrengues et al. (2018)
Fc receptor inhibitor	Fostamatinib	Disrupt FcγR-mediated NETosis Decrease circulating NETs in COVID-19	In vitro Clinical trials	Strich et al. (2021a, b); Strich, Tian et al. (2021)
NADPH oxidase inhibitor	Diphenyleneiodonium	Reduce lytic NETosis	In vitro	Fuchs et al. (2007)
NADPH oxidase inhibitor	Apocynin	Reduce lytic NETosis	In vitro Clinical trials	Stefanska et al. (2012); Takesue et al. (2020)
PKC inhibitor	Metformin	Reduce lytic NETosis	In vitro Clinically used	Batchuluun et al. (2014); Menegazzo et al. (2018)
NE inhibitor	Sivelestat	Reduce lytic NETosis	In vitro Clinical trials	Makino et al. (2011); Okeke et al. (2020); Zhou et al. (2020)
NE inhibitor	GW311616A	Reduce lytic NETosis	In vitro	Papayannopoulos et al. (2010)
PAD4 inhibitor	GSK484 Chloramidine (Cl-amidine)	Reduce NETosis and NET-related tumor growth	In vivo In vitro	Albrengues et al. (2018); Li et al. (2010); Park et al. (2016); Teijeira et al. (2020); Xiao et al. (2021)
GSDMD antagonist	LDC7559	Reduce lytic NETosis	In vitro	Sollberger et al. (2018)
Cathepsin C inhibitor	AZD7986	Inhibit cathepsin-induced NETosis and reduce lung metastasis of breast cancer	In vivo	Xiao et al. (2021)
DNase	–	Degrade NETs backbone and decrease tumor progression	In vivo	Cools-Lartigue et al. (2013); Miller-Ocuin et al. (2019); Nie et al. (2019); Park et al. (2016); Takesue et al. (2020); Teijeira et al. (2020); Tohme et al. (2016); Yang et al. (2020a, b)
TLR4 antagonist	Eritoran	Reduce NET-related mitochondrial biogenesis in murine colon adenocarcinoma cells	In vitro	Yazdani et al. (2019)
TLR9 antagonist	Oligonucleotide	Reduce NET-induced proliferation in lymphoma cells	In vitro	Nie et al. (2019)
VWF protease	ADAMST13	Reduce VWF-dependent NET adherence to the vascular wall and minimize NET-related damage to liver	In vivo	Kolaczkowska et al. (2015)
Unfractionated heparin	–	Accelerate NET degradation by suppressing VWF-NETs interaction	In vitro	Grässle et al. (2014); Leppkes et al. (2020)
COX-1 inhibitor	Aspirin	Impair NET formation by impeding NF-κB activation	In vitro	Lapponi et al. (2013)
COX-2 inhibitor	Celecoxib	Reduce NET-enhanced invasion capacity of HCC cells	In vitro	Yang et al. (2020b)

*NET *neutrophil extracellular trap,, *CXCR* CXC chemokine receptor, *LPS* lipopolysaccharide, *PKC* protein kinase C, *PAD4* protein arginine deiminase 4, *GSDMD* gasdermin D, *TLR* toll-like receptor, *VWF* von Willebrand factor, *COX* cyclooxygenase, *HCC* hepatocellular carcinoma

### The inhibitors of NET formation

Targeting first messengers and their receptors is a widely used therapeutic strategy, which can also be applied to reduce NET formation and NET-mediated changes. IL-8 secreted by tumor cells induces lytic NETosis via the CXCR2-mediated pathway. Treatment with reparixin, an inhibitor of CXCR1 and CXCR2, largely decreases the intra-tumoral NET formation in breast cancer-bearing mice (Teijeira et al. 2020). Another study also showed that CXCR2 inhibition significantly reduced NET-primed tumor growth in lymphoma-bearing mice (Nie et al. 2019). More proof was obtained in studies on infectious diseases and chronic inflammatory conditions, in which C–X–C chemokine receptor antagonists successfully inhibits NETosis and alleviates inflammation (Pedersen et al. 2018). Another mechanism of lytic NETosis was mediated by immune complex and Fcγ receptor. Fostamatinib, a FDA-approved oral agent for the treatment of chronic idiopathic thrombocytopenic purpura, is able to disrupt FcγR-mediated signaling and inhibit NET formation in vitro (Strich et al. 2021a, b). Moreover, a phase II study targeting COVID-19 showed that plasma NETs decreased more rapidly in patients receiving fostamatinib compared with those receiving placebo (Strich, Tian, et al. 2021). Besides tumor-derived cytokines and immune complexes, several PAMPs and DAMPs, such as LPS and HMGB1, are considered potent inducers of NOX-independent NETosis. Taurolidine, an antimicrobial drug which denatures LPS, is able to decrease tobacco smoke-induced NET formation and attenuate NET-related activation of quiescent cancer cells (Albrengues et al. 2018). Similarly, the administration of anti-HMGB1 antibodies effectively decreases NET formation in vitro and in vivo (Kim et al. 2019; Tadie et al. 2013).

ROS is an essential second messenger involved in classical NET formation. Neutrophils isolated from patients with chronic granulomatous disease (CGD), a disease that renders patients unable to produce enough ROS due to NADPH oxidase deficiency, fail to release NETs if they are stimulated with PMA or *S. aureus* (Fuchs et al. 2007). Likewise, the inhibition of NADPH oxidase by diphenyleneiodonium (Fuchs et al. 2007) or apocynin (Takesue et al. 2020), dramatically decreases NET formation in vitro. Although the clinical validity of these NADPH inhibitors is largely unknown, the biosafety and pharmacokinetics of setanaxib (oral administration) and apocynin (nebulization) have been confirmed in phase I and phase II studies (Elbatreek et al. 2020; Stefanska et al. 2012). Metformin, a first-line medication for diabetes, inhibits the PKC-NADPH oxidase axis by reducing the membrane translocation of PKC-βII (Batchuluun et al. 2014). Metformin was shown to completely block PMA-induced NETosis in vitro. In patients who received metformin therapy, the concentration of plasma NET biomarkers, including cell-free DNA, NE, PR3, were significantly reduced (Menegazzo et al. 2018).

NE and PAD4 are two key enzymes which regulate NET formation. Both enzymes contribute to histone modification and chromatin decondensation. In addition to histone cleavage, NET-bound NE may interact with target cells to provoke a pro-inflammatory or procoagulant state. As such, the administration of NE inhibitors sivelestat, not only prevents NETosis, but also limits NET-mediated cytotoxicity and response (Okeke et al. 2020; Zhou et al. 2020). Sivelestat has been clinically tested in thoracoscopic esophagectomy for esophageal cancer and successfully reduced the incidence of post-operative acute lung injury and decreased surgery-induced pulmonary function loss (Makino et al. 2011). As mentioned before, PAD4 is another essential enzyme involved in NETosis. PAD4 knockout mice are widely used as models to study the impact of the absence of NETs. Without the presence of NETs, tumors often grow slower in PAD4 knockout mice compared to wild type controls (Yazdani et al. 2019). Similarly, the pharmacological inhibition of PAD4 with chloramidine (Li et al. 2010; Park et al. 2016) or GSK484 (Teijeira et al. 2020) also reduces NET production and ameliorates the disease progression in various models of autoimmune and infectious disease.

Although pre-clinical models have proven the effectiveness of the NET formation inhibitors mentioned above, it is worth noting that many of the targets, such as NE and NADPH oxidase, are integral components of neutrophils’ antibacterial functions. It remains to be studied whether inhibiting these enzymes increases the risk of infection.

### Blocking NET-mediated action

DNAse could effectively remove NETs by degrading their DNA backbones. Several in vivo studies have demonstrated that DNase treatment could prominently reduce NET-mediated tumor growth and metastasis (Cools-Lartigue et al. 2013; Miller-Ocuin et al. 2019; Nie et al. 2019; Park et al. 2016; Takesue et al. 2020; Teijeira et al. 2020; Tohme et al. 2016; Yang et al. 2020a, b). For example, intravenous administration of DNAse could minimize sepsis-induced NETs and attenuate hepatic metastases in vivo (Cools-Lartigue et al. 2013; Tohme et al. 2016). The biosafety of DNase has already been tested in clinical trials for indications other than cancer. Dornase alfa is a commercially available recombinant human DNase. Nebulization of dornase alfa ameliorates the disease progression of cystic fibrosis by reducing the viscosity of the sputum and reduces post-operative lower respiratory tract infection in patients receiving lung transplants (Tarrant et al. 2019). NETs are frequently found in various pathological conditions of the lower respiratory tract (Hudock et al. 2020; Pandolfi et al. 2021; Tadie et al. 2013; Zhang et al. 2020b, the inhalation of DNase could be a solution to reduce NET-related tissue damage and minimize local inflammation. Moreover, some clinical trials tested the use of DNase as adjuvant therapy against leukemia (NCT02462265) or head and neck cancer (NCT00536952). Oklu et al*.* suggested that the reduced levels and activities of endogenous nuclease were responsible for increased NETs in the circulation of cancer patients (Oklu et al. 2017). Therefore, DNase supplementation could be an efficient therapeutic strategy to reduce tumor-associated NET formation and NET-mediated pro-metastatic responses.

TLRs are the most studied receptors involved in NET recognition and signal initiation. The effects of NETs may diminish when target cells are transfected with TLR siRNA or treated with specific antagonists. For examples, silencing of TLR2 attenuates NET-mediated endothelial cell activation (Quillard et al. 2015), blockage of TLR4 by siRNA and Eritoran reduces mitochondrial biogenesis in MC38 colon adenocarcinoma cells (Yazdani et al. 2019), blockage of endosomal TLR9 by oligonucleotide decreases the NET-induced proliferation in SU-DHL-2 lymphoma cells (Nie et al. 2019). NET-mediated pro-inflammatory response largely vanishes in human hepatocellular cells when TLR4 and TLR9 are both knocked out (Yang et al. 2020b). Although a series of in vitro experiments have proved that TLRs could suppress NET-mediated actions, more studies need to be done to evaluate the effectiveness of TLR inhibition in vivo.

Von Willebrand factor (VWF) is an adhesive glycoprotein expressed on endothelial cells and Kupffer cells (Wong et al. 2013). Some studies have demonstrated that VWF serves as a binding site for the DNA component of NETs, which allows the attachment of NETs to the lumen of vessels (Grässle et al. 2014; Kolaczkowska et al. 2015). Intravascular administration of ADAMST13, a VWF protease, reduces VWF-dependent NET adherence to the vascular wall and minimizes their damage to liver (Kolaczkowska et al. 2015). Recombinant ADAMTS-13 has been shown to be safe and well-tolerated in clinical trials for hereditary thrombotic thrombocytopenic purpura (Scully et al. 2017), so it could perhaps be used to remove intravascular NETs and diminish NET-mediated tumor cell adhesion in the future. Furthermore, unfractionated heparin could strongly accelerate NET degradation in vitro by suppressing VWF-NETs interaction (Grässle et al. 2014; Leppkes et al. 2020), whilst low-molecule-weight heparin was less effective in clearing NETs (Grässle et al. 2014). The appropriate administration of unfractioned heparin may reduce the NETosis caused by surgical interventions and benefit the patients.

Aspirin is one of the most commonly used anti-inflammatory and anti-platelet agents. The anti-platelet effect of aspirin may protect patients against metastasis and relapse. In our previous study, we found that perioperative aspirin intake significantly prolonged the disease-free period in patients who received curative resection for pancreatic cancer (Pretzsch et al. 2021). Interestingly, in vitro and in vivo models also show that aspirin could impair NET formation by impeding NF-κB activation (Lapponi et al. 2013). As we discussed above, the interaction between NETs and platelets is bi-directional. Activated platelets could be a potent inducer of NETosis, and NETs in turn could further enhance the activation and pro-metastatic actions of platelets. Thereby, the use of aspirin may prevent both NET formation and NET-platelet interactions. Unlike other NASIDs, aspirin has a weak inhibitory role on COX-2, a key enzyme involved in NETs-mediated inflammatory response in HCC (Yang et al. 2020b) and breast cancer (Martins-Cardoso et al. 2020). Celecoxib, a selective COX-2 inhibitor, could reduce the NET-enhanced invasion capacity of HCC cells in vitro (Yang et al. 2020b). More research is still needed to determine how NSAIDs interact with NETs, and whether or not they can be effectively used against NETs in routine practice.

Certain medical interventions may increase the risk of developing NETosis and cause NET-related adverse effects. Granulocyte-colony stimulating factor (G-CSF) injection is a standard treatment for hematopoietic stem-cell transplantation and chemotherapy-induced neutropenia. G-CSF not only stimulates the proliferation of neutrophils, but also induces them to release NETs (Demers et al. 2016; Giaglis et al. 2016; Schoergenhofer et al. 2017). NET formation induced by G-CSF treatment significantly promotes tumor growth in vivo (Demers et al. 2016). Another example is radiotherapy in bladder cancer. Shinde-Jadhav et al*.* found that HMGB1 released during radiation eventually led to NET formation, which may contribute to radiation resistance (Shinde-Jadhav et al. 2021). Besides medical treatment, surgical operations also predispose NET formation (Beaubien-Souligny et al. 2019; Cools-Lartigue et al. 2013; Ren et al. 2021; Tohme et al. 2016; von Meijenfeldt et al. 2018). Curative surgery is the frontline therapy for patients with resectable tumors, however, relapses and distant metastases are common even after an uneventful operation. The NETs formed during the surgery may create a pre-metastatic niche which favors the implantation of circulating tumor cells (Cools-Lartigue et al. 2013; Ren et al. 2021; Tohme et al. 2016). Several in vivo studies showed intravenous administration of DNase could diminish surgical-related NET deposition and attenuate the distant metastases (Cools-Lartigue et al. 2013; Tohme et al. 2016). Prophylactic use of NET inhibitors, such as DNase, may reduce undesired effects caused by NETs and improve therapeutic outcomes.

## Conclusion

Neutrophils release NETs as an innate immune response to various infectious and inflammatory stimuli. Although one of the main purposes of NET formation is to counter microbial invasion, it also plays pathological roles in tumorigenesis and metastasis. NETs consist of various damage-associated molecular patterns, which can be recognized by a series of pattern recognition receptors and initiate downstream signaling. There is plenty of evidence that NETs interact directly with cancer cells. These interactions may promote tumor cell proliferation, induce epithelial–mesenchymal transition, facilitate adhesion of circulating tumor cells or awaken dormant cancer cells. The evidence also showed that NETs might modulate the tumor microenvironment by degrading anti-tumorigenic ECM elements, activating cancer-associated fibroblasts and inhibiting the cytotoxicity of tumor-associated immune cells. The deposition of NETs in distant organs may create a pre-metastatic niche to host the circulating tumor cells, and thus promote distant metastasis. Circulating NET markers, including H3Cit and MPO-dsDNA, although not specific for cancer-associated NET formation, might be useful to detect relevant NETosis and thus high risk of metastasis. Although the pro-tumorigenic roles of NETs have been widely recognized, therapeutic strategies targeting NETs still need to be developed. Several NET inhibitors, including DNase, have been tested in vivo or in early clinical trials for other indications. Considering the role of NETs in tumor progression, targeting NETs could be a promising diagnostic and therapeutic approach for cancer management.
